# Competition Increases Sensitivity of Wheat (*Triticum aestivum*) to Biotic Plant-Soil Feedback

**DOI:** 10.1371/journal.pone.0066085

**Published:** 2013-06-12

**Authors:** W. H. Gera Hol, Wietse de Boer, Freddy ten Hooven, Wim H. van der Putten

**Affiliations:** 1 Netherlands Institute of Ecology, Department of Terrestrial Ecology, Wageningen, The Netherlands; 2 Netherlands Institute of Ecology, Department of Microbial Ecology, Wageningen, The Netherlands; 3 Wageningen University, Department of Soil Quality, Wageningen, The Netherlands; 4 Wageningen University, Laboratory of Nematology, Wageningen, The Netherlands; University of Alberta, Canada

## Abstract

Plant-soil feedback (PSF) and plant competition play an important role in structuring vegetation composition, but their interaction remains unclear. Recent studies suggest that competing plants could dilute pathogenic effects, whereas the standing view is that competition may increase the sensitivity of the focal plant to PSF. In agro-ecosystems each of these two options would yield contrasting outcomes: reduced versus enhanced effects of weeds on crop biomass production. To test the effect of competition on sensitivity to PSF, we grew *Triticum aestivum* (Common wheat) with and without competition from a weed community composed of *Vicia villosa*, *Chenopodium album* and *Myosotis arvensis*. Plants were grown in sterilized soil, with or without living field inoculum from 4 farms in the UK. In the conditioning phase, field inocula had both positive and negative effects on *T. aestivum* shoot biomass, depending on farm. In the feedback phase the differences between shoot biomass in *T. aestivum* monoculture on non-inoculated and inoculated soils had mostly disappeared. However, *T. aestivum* plants growing in mixtures in the feedback phase were larger on non-inoculated soil than on inoculated soil. Hence, *T. aestivum* was more sensitive to competition when the field soil biota was present. This was supported by the statistically significant negative correlation between shoot biomass of weeds and *T. aestivum*, which was absent on sterilized soil. In conclusion, competition in cereal crop-weed systems appears to increase cereal crop sensitivity to soil biota.

## Introduction

Plants have selective effects on soil biota, with negative, neutral or positive consequences for future occupants of the same location [1], [2]. These biotic plant-soil feedback (PSF) effects are caused by net effects of soil-borne mutualists and pathogens that develop in the rhizosphere during plant growth [3]. In addition, there are abiotic feedbacks such as changes in nutrient or water availability. If the performance of conspecific plants in the next growing cycle is stimulated, this is called a positive PSF. If conspecific plants perform worse in the next growing cycle, this is called a negative PSF. This process is important for plant succession [4], [5] and diversity-productivity relationships [6], [7], but it also plays an important role in applied areas, such as agriculture [8], restoration ecology [9], [10] and invasion management [11]. The success of later successional plants traditionally has been attributed to their ability to constrain growth of predecessors [12], but more recently it has been acknowledged that species-specific PSF can influence predecessor-successor interactions [1], [13], [14].

In agriculture, crop species are being grown in rotation in order to avoid the development of negative PSF, as crop rotation prevents the accumulation of crop-specific soil pathogens [8]. Negative intraspecific PSF effects can be avoided by growing crops in successional cycles with other crops that are not sensitive to the pathogens from previous crops. The crop cycles can also be related to using positive interspecific PSF, for example green manures that increase nutrient availability and nitrogen-fixing plants that enhance the pools of available nitrogen in soil. Intercropping with legumes has been used to both reduce pathogen pressure (negative PSF) and simultaneously increase soil nitrogen (positive PSF) [15].

Empirical work on PSF has been done both with single plant species and with communities [2], mostly composed of wild plants. Shifts in relative abundance of plant species within a community indicate that PSF does affect competitive relations, e.g [5], but only a few studies have addressed the role of competition in PSF studies explicitly [1], [13].

There are two mechanisms in relation to feedback in plant mixtures which would lead to opposite effects. Plants that are stressed due to competition can be more vulnerable to pathogens [16]. Successful competitors may need to invest in longer stems to compete for light or larger roots to compete for nutrients and these investments may come at the expenses of plant defense [17]. On the other hand, pathogen effects can be density-dependent and since the relative abundance of species in mixtures is lower than in monocultures less pathogenic effects can be expected and observed in mixtures [6], [7].

Thus far there is limited evidence that intraspecific competition increases PSFs [2] or that interspecific competition decreased PSF [18]–[20]; but see [1], [21]–[23]. We analyze the effect of competition on plant soil feedback of wheat in the feedback phase. Competition can be studied with replacement (constant density) or additive designs (increasing density) [24]. We choose an additive design without the option to distinguish intra- from interspecific competition since this resembles the agricultural situation best. The outcome of the interaction between feedback and competition may depend on species identity [25], and thus it is important to use species which co-occur in order to provide relevant information for a particular system. All soils were conditioned with *T. aestivum* (common wheat), and nutrients were added to minimize the role of nutrient availability on plant growth responses and to focus on biotic feedback [23], [26]. We determined how the presence of a weed community affects responses of common wheat (*Triticum aestivum*) to PSF, by performing a feedback experiment with monoculture and plant mixtures in the feedback phase. For our experiment soil was collected from 4 different locations to determine whether the effect of competition on PSF is general or rather depends on the specific soil biota. Given the high density of crop plants in agriculture, we expect that increased sensitivity to pathogens will overrule the reduction in pathogen density dependence effects by weeds. Thus we hypothesize that negative plant-soil feedback effects of *T. aestivum* plants will be enhanced when *T. aestivum* grows in plant mixtures. Our results show that plants with competition are more vulnerable to negative plant soil feedback effects.

## Materials and Methods

The effect of plant mixtures on sensitivity to plant soil feedback was tested by growing *Triticum aestivum* L. plants for two growing cycles in the same soil, in the presence or absence of arable weeds in the second growing cycle. The first growing cycle consisted of wheat plants growing in sterilized soil with or without field soil inoculum. The aim of this phase was to standardize abiotic conditions and determine the variation in effects of soil biota from different farms on wheat growth. Since all soils were conditioned with *T. aestivum*, we minimized the role of soil nutrient feedback and focused on the biotic feedback.

### Soils

Soil for the experiment was collected with approval from the owners of 4 farms from the Chilterns area (United Kingdom). The soil-type is chromic luvisol/leptosol with pH in the range of 7.2–7.6. At each farm, soil from three fields was collected. Soil was passed through a 1 cm sieve to remove stones and reduce the larger clumps of clay. For each farm 66 kg soil (a mix from the 3 fields) was sterilized by gamma radiation (25 kGray, Isotron, Ede, The Netherlands) in order to create a sterilized background soil. A small subsample from each field was kept apart and remained non-sterilized in order to serve as an inoculum for soil biota. The sterilized soil was used to fill square pots (11×11×12 cm) with 1 kg soil, including 90 g fresh living soil inoculum (9% inoculum). Eight pots per field were filled, resulting in 4 (farms) * 3 (fields) * 8 (pots) = 96 pots in total. In addition to the aforementioned treatments of sterilized soil with non-sterilised soil inoculum, a control treatment was included which consisted of 16 pots per farm filled with sterilized soil only. This added 4 (farms) * 16 (pots) = 64 pots to the experiment. The amount of replicates for the control pots was doubled from 8 to 16 to increase the balance when comparing inoculated vs non-inoculated pots.

### Conditioning phase

For the first phase of the experiment, all pots were sown with 7 seeds of common wheat (*Triticum aestivum*) in a row in the middle of the pots. There were 24 inoculated (3 fields * 8 replicates) and 16 non-inoculated control pots per farm. Plants were kept under controlled conditions in the greenhouse (60% RH; 16 h L, 8 h D, 21°C/16°C) and placed in random order on a bench. Additional illumination was provided by 400W growing bulbs (Philips SON-T Agro, Philips, Eindhoven, The Netherlands). Light intensity at plant level was 225 µmol PAR. Pots were watered regularly with demineralized water and received 60 ml week^−1^ Hoagland solution (half strength, [27]). Nutrient addition may alter feedback effects [28], but was chosen to reduce possible nutrient feedback effects and focus on the biotic component. In addition, fertilization is common practice in agricultural systems. A thrips infestation was controlled by the predatory mite *Amblyseius cucumeris* (Koppert Biological Systems, Berkel en Rodenrijs, The Netherlands). Plants were harvested after 60 days; aboveground biomass was clipped and put in paper bags. Shoot dry weight was determined after 72 h drying at 70°C.

### Feedback phase

For the second phase of the experiment, soil with roots was left in the pots and re-growing shoots were removed. After two weeks no more re-growth was observed and the pots were sown again with 7 seeds of *T. aestivum*. Seeds were sown perpendicular to the previous sown seeds, to be able to discriminate between potential re-growth and germination, but this precaution proved unnecessary. Half of the pots was planted with one seedling from each of the three weed species: hairy vetch (*Vicia villosa* Roth), lamb's quarters (*Chenopodium album* L.) and field forget-me-not (*Myosotis arvensis* (L.) Hill.). Seeds from *C. album* and *V. villosa* were collected in the wild in the Netherlands, while *M. arvensis* was ordered from a commercial supplier (Rieger-Hofmann, Blaufelden-Raboldshausen, Germany). Seeds were germinated on glass pearls by 16 h L, 8 h D, 23°C/15°C. *Chenopodium album* and *M. arvensis* are common arable weeds [29]; *Vicia villosa* is often applied as winter cover crop and might return as weed in follow-up crops. The weeds resulted in 2 treatments in the second phase: Mono and Mix (n = 12 for inoculated pots and n = 8 for non-inoculated control pots). The pots were kept in the same greenhouse as before, under the same light and temperature conditions. Pots were watered regularly with demineralized water and received 60 ml week^−1^ Hoagland solution (half strength, [27]). After 55 days the aboveground biomass was harvested as described above for the conditioning phase. Shoots from all pots were separated per species and dry weight per species per pot was determined after 72 h drying at 70°C. For a subset of plants (farm 3) the shoot material was ground in a Retsch mill and carbon and nitrogen was measured in three milligrams of the leaves by combustion with an elemental autoanalyzer Flash EA 1112 NC analyzer (Interscience, Breda, the Netherlands) to determine whether inoculation affected nitrogen and carbon content.

### Statistical analyses

Differences in shoot biomass of *T. aestivum* on inoculated and non-inoculated soils in the conditioning phase were tested with a linear model: shoot biomass ∼ Inoculum * Farm. Farm was included as fixed effect since we were interested in the generality of the tested responses. Normality of the residuals was tested with the non-parametric Kolmogorov-Smirnov test and Homogeneity of variances with the non-parametric Fligner test. Residuals were not normally distributed and this could not be improved by transformation or removal of outliers. However, non-parametric tests (Wilcoxon test per farm) gave the same results as the linear model and thus violation of the assumptions appeared not too influential. For consistency with the analyses in the feedback phase, we decided to present the linear model results. In the feedback phase we compared shoot biomass of *T. aestivum* between inoculated and non-inoculated soils with a linear model using Inoculation, Farm and Competition as fixed categorical factors. Model check plots were made to inspect behavior of the residuals and normality of errors; no transformations were necessary. We also run an additional model where weed biomass was included as a covariate to verify whether this could explain possible Inoculation effects. When covariates were significant, correlations were tested with Spearman's rank correlation tests. This was done separately for the two treatments in the feedback phase (inoculated and non- inoculated). Difference in nitrogen percentage in the shoots of plants growing on inoculated and non-inoculated soils was tested with a linear model nitrogen ∼ Inoculum * Competition and verified non-parametrically with a Wilcoxon test. Since the interaction Inoculum * Competition was not significant (P = 0.96) we only present the main fixed effects for the model nitrogen ∼ Inoculum + Competition. All statistical analyses were carried out with R 2.14.1 statistical package [30]. The data has been archived in the Data and Information Portal of the NIOO-KNAW http://data.nioo.knaw.nl/index.php and is available on request.

## Results

### Conditioning phase

**Figure 1:**
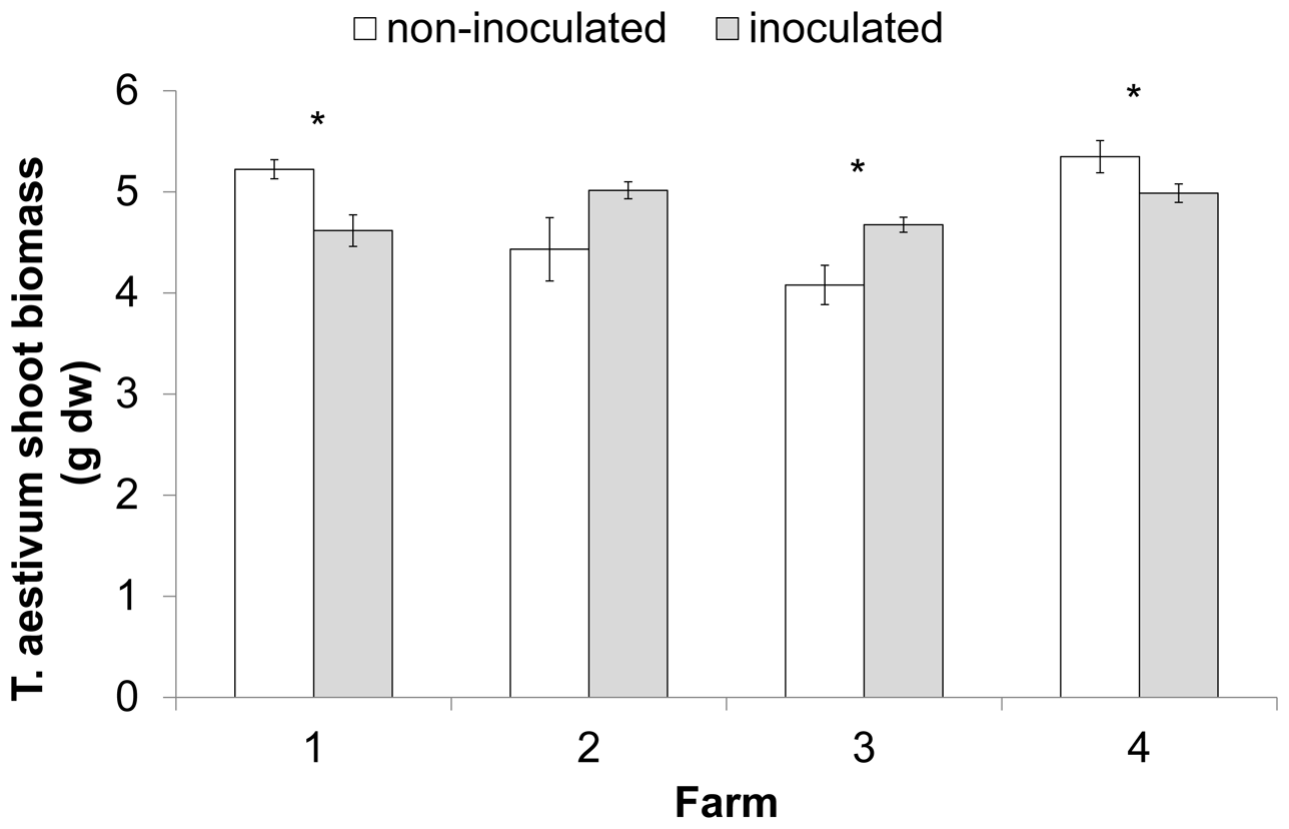
Soil inoculation and plant biomass. Shoot biomass of *Triticum aestivum* (mean ± SE) on sterilized soil without inoculum or with 9% field soil inoculum from 4 different farms in the conditioning phase. The asterisks indicate significant differences (*P*<0.05) between non-inoculated and inoculated soil.

The effect of inoculation with field soil on *T. aestivum* shoot biomass varied between farms (Fig. 1, Table 1). For two farms inoculated soil yielded more *T. aestivum* shoot biomass (10%±3) than non-inoculated soil, whereas the other two farms showed the opposite pattern (−12%±0.5).

**Table 1:** Linear model testing effect of inoculation and farm on shoot biomass of *Triticum aestivum* in the conditioning phase.

	Estimate	Std. Error	*t*-value	*P*-value
Intercept	4.61	0.13	34.45	<0.01
Inoculation	0.61	0.21	2.86	<0.01
Farm 2	0.40	0.19	2.10	0.04
Farm 3	0.06	0.19	0.31	0.76
Farm 4	0.37	0.19	1.95	0.05
Inoculation*Farm 2	−1.19	0.30	−3.97	<0.01
Inoculation*Farm 3	−1.20	0.30	−4.01	<0.01
Inoculation*Farm 4	−0.25	0.30	−0.82	0.41

### Effect of crop-weed mixtures on vulnerability to PSF

**Figure 2:**
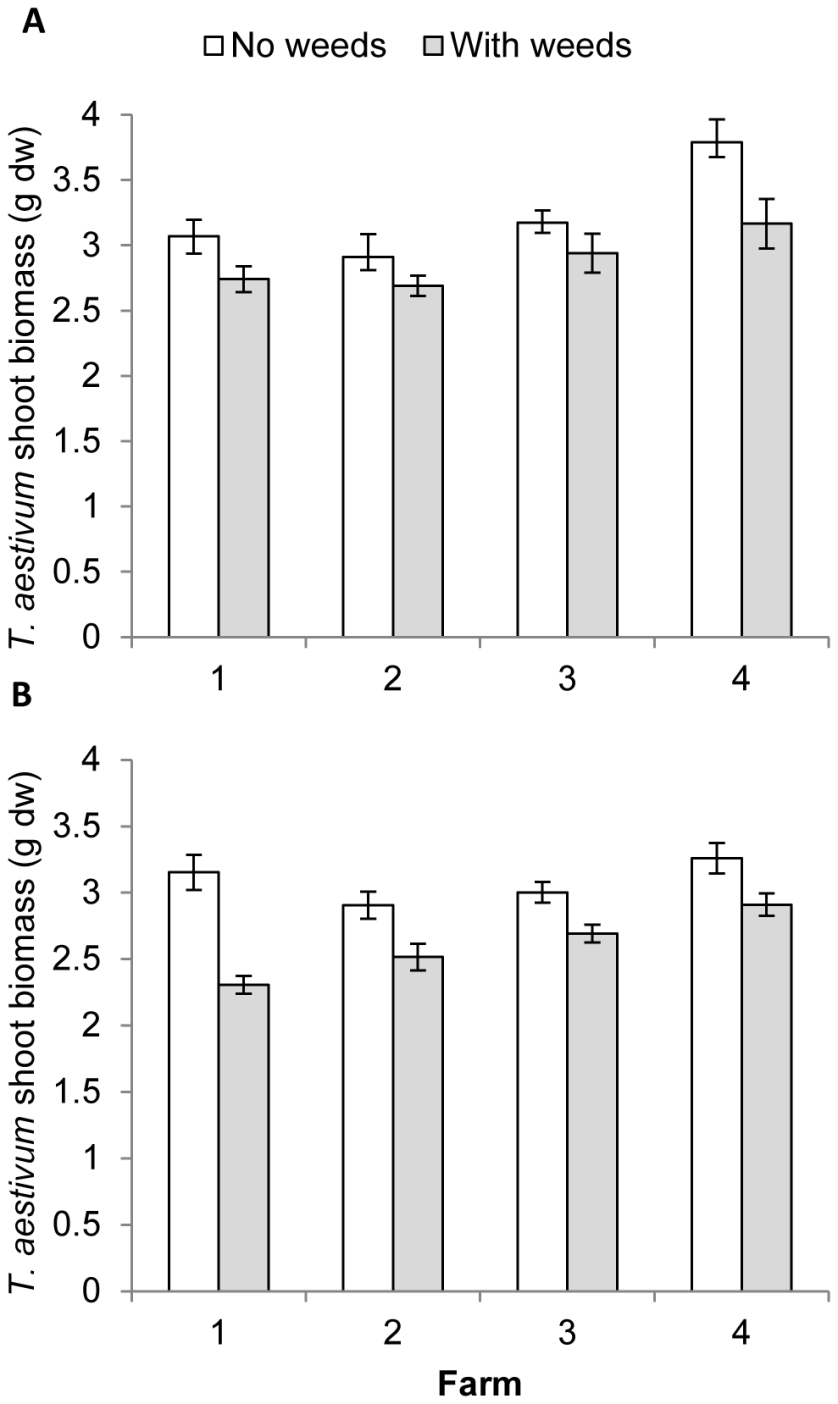
Competition interacts with inoculation effects. Shoot biomass of *Triticum aestivum* (mean ± SE) growing in soil with or without weeds. **A** growing in sterilized soil, **B** growing in inoculated soil.

**Figure 3:**
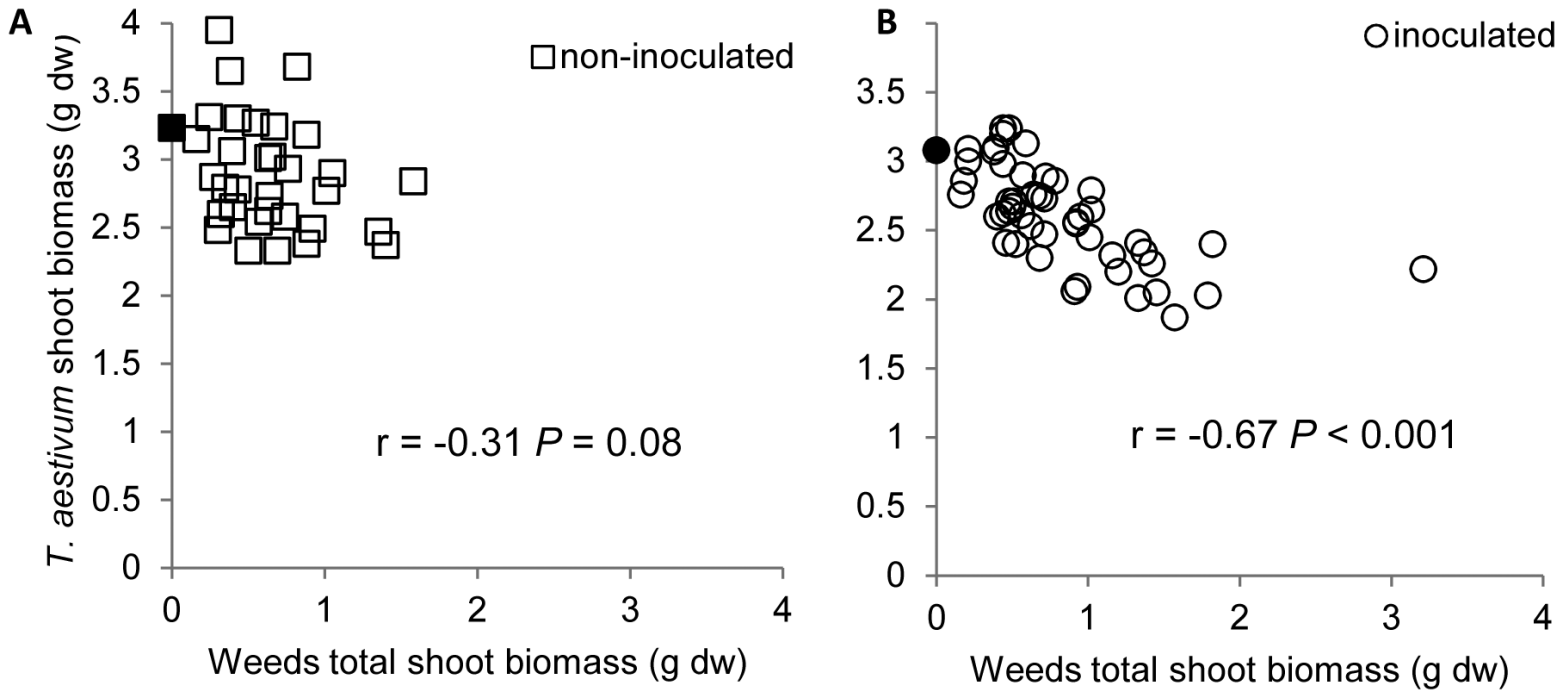
Correlation between crop and weeds depend on soil inoculation. Correlation between *T. aestivum* shoot biomass and weeds shoot biomass within the same pot on sterilized **A** non-inoculated soil and **B** inoculated soil in the feedback phase. Spearman rank correlation coefficients and *P*-values are shown in the graphs. Filled symbols indicate the average *T. aestivum* shoot biomass in the absence of weeds; n = 32 for non-inoculated soils and n = 48 for inoculated soils.

In the feedback phase the effect of inoculation interacted with farm and competition (significant 3-way interaction, Table 2). The presence of weeds reduced *T. aestivum* shoot biomass in all cases (Fig. 2A, 2B), but the strength of the effect is modified by farm and inoculation. On farms 1, 2 and 3 the growth reduction by competition was smaller on sterilized soil (7–10%, Fig. 2A) than on inoculated soil (10–27%, Fig. 2B). Farm 4 is the exception, with stronger growth reduction by competition on the sterilized soil. Since total weed biomass was not significantly affected by inoculation, the difference in *T. aestivum* shoot biomass between sterilized and inoculated soils is probably not due to weeds being smaller on non-inoculated soil. When weed biomass was included as covariate, Inoculum is retained as a, albeit marginally, significant factor in the model. Apparently *T. aestivum* is more sensitive to competition when the field soil biota was present. Further support for this hypothesis can be found when considering the relation between shoot biomass of weeds and *T. aestivum*. For sterilized soil there is no correlation between those two (Fig. 3A), while for inoculated soil this correlation is significantly negative (Fig. 3B).

**Table 2:** Linear model testing effect of inoculation, competition and farm on shoot biomass of *Triticum aestivum* in the feedback phase.

	Estimate	Std. Error	*t*-value	*P*-value
Intercept	3.15	0.10	30.32	<0.01
Inoculation	−0.08	0.16	−0.51	0.61
Farm 2	−0.25	0.15	−1.69	0.09
Farm 3	−0.15	0.15	−1.02	0.31
Farm 4	0.11	0.15	0.72	0.47
Competition	−0.85	0.15	−5.76	<0.01
Inoculation*Farm 2	0.09	0.23	0.38	0.70
Inoculation*Farm 3	0.25	0.23	1.08	0.28
Inoculation*Farm 4	0.61	0.23	2.63	<0.01
Competition*Farm2	0.46	0.21	2.20	0.03
Competition*Farm3	0.54	0.21	2.58	0.01
Competition*Farm4	0.50	0.21	2.40	0.02
Inoculation*Competition	0.52	0.23	2.22	0.03
Inoculation*Farm 2*Competition	−0.35	0.33	−1.06	0.29
Inoculation*Farm 3* Competition	−0.44	0.33	−1.33	0.19
Inoculation*Farm 4* Competition	−0.79	0.33	−2.40	0.02

The presence of biota appeared to have increased either nutrient availability or uptake, since nitrogen percentage in the shoot during the feedback phase was slightly higher for inoculated soils (1.60±0.05% for inoculated soils, n = 23, 1.42±0.06% for non-inoculated soils, n = 16, lm Inoculum *P* = 0.02, Competition *P* = 0.31, no significant interaction). Legumes are known for increasing nitrogen, but in our experiment the presence of weeds including one legume species did not significantly affect nitrogen or carbon percentages in the shoot of *T. aestivum*.

## Discussion

### Effect of crop-weed mixtures on vulnerability to PSF

Plant mixtures in the feedback phase showed reduced wheat focal shoot biomass, which might be a combination of interspecific competition and enhanced sensitivity to plant soil feedback. In monoculture, however, there was very little evidence of plant soil feedback deviating from neutral. Negative effects of soil inoculation on shoot biomass in the conditioning phase may represent net pathogenic effects of soil biota, which might be even stronger in the feedback phase since the pathogens accumulated during conditioning. The same could be expected for the positive effects, unless positive biota need more time to establish, but in fact soils from only 1 out of 4 farms showed a repeated pattern of the conditioning phase in the feedback phase. Plants in soil from Farm 4 always grew better in sterilized soil compared to inoculated soil. Plants in soil from Farms 1, 2 and 3 did equally well on inoculated and non-inoculated soil in the feedback phase, despite the differences in seen in the conditioning phase. Therefore, regardless of the legacy effects in the conditioning phase, there was little difference between inoculated and non-inoculated soils for the monoculture treatment in terms of biomass and only the nitrogen data indicated that there is some biotic feedback. Feedback effects became more apparent under competition; addition of weed seedlings to half of the pots in the feedback phase led to consistently smaller *T. aestivum* plants on inoculated soils as compared to the non-inoculated ones. Likewise, Callaway et al. [22] only found effects of soil biota on *Centaurea maculosa* in greenhouse experiments when competitors were present. Stronger negative feedback effects under competition have been found before, in experiments with replacement designs [13], [18], [19], where total plant density is kept constant. Manipulation of soil communities independent of plant communities showed that overyielding was due to plants inhibited by their own soil biota and unrelated to nutrient availability [31]. Shannon et al. [20] also used an additive design and found no evidence for feedback when plants were grown separately; only in competitive mixtures did feedback become apparent. Yet there is no consistency in the relationship between competition and feedback; feedback effects might also disappear under conditions of competition [22] and in some circumstances feedback effects might override competition effects [23]. The variety in effects might be related to soil origin and to plant species identity [22], although a study with 24 species showed that the majority of species suffered stronger negative feedback when grown in competition [19]. Even without competition plant soil feedbacks have been described as idiosyncratic [32], [33] often meaning unpredictable [34] and the challenge is to find generalities in plant-soil feedback. The added value of the present study is the demonstration that biotic feedback effects were increased when co-occurring species compete and this was found across a wide range of farms and fields.

On non-inoculated soils *T. aestivum* plants had larger shoots and appeared to be less affected by competing weeds than on the inoculated soil. One possible explanation would be that the weeds did not grow well on the non-inoculated soil. However, weed biomass was not significantly different between non-inoculated and inoculated soils and including weed biomass as a covariate did not affect the significant difference between control soil and the inoculated soils. The degradation of wheat roots might also have differed between inoculated and non-inoculated soils, with consequences for plant nutrition. Since we made no measurements of plant growth during the experiment, we cannot exclude the possibility that there were initially differences in *T. aestivum* monocultures between sterilized non-inoculated and inoculated soil which disappeared in the later growth phase. This has been found before [13] and in plant mixtures the weeds could have taken advantage of the temporary growth delay of the wheat plants, thus effectively fixating the difference. This would also result in larger wheat plants in the feedback phase on sterilized control soils than on the inoculated soils. However, if weeds used the window of opportunity created in inoculated soil, then larger weed biomass would be expected for inoculated soil than for the sterilized control soils, but this was not found. Thus the most parsimonious interpretation is that *T. aestivum* plants growing in mixtures were more sensitive to their biotic PSF, e.g. due to a trade-off between resources allocated to either competitive or defense traits.

A possible mechanism for increased effects of PSF under competition could be the increased amount of roots in pots with plant mixtures. Root density is an important factor in development of fungal diseases [35]. However, the question is whether this would also work for specific pathogens. Generally, increasing plant diversity is thought to dilute species-specific pathogens [6], [7]. The fact that the current study reveals that *T. aestivum* growing in plant mixtures was more sensitive to PSF might depend on the dominance of *T. aestivum*. This should be tested in a PSF experiment where the focal plant density is kept constant and the competitors are added in a large range to test whether there is a tipping point from increased negative effects of PSF in mixtures towards a dilution of plant-specific pathogens. Aguilera [36] modeled two-species competition under a range of competitive abilities and feedback scenario's and demonstrated that feedbacks can reverse the outcome of competition. The existence of such a tipping point will depend on the strength of the PSF versus the competitive effects. Knowledge of such a point could be used to determine optimal planting densities in mixed cropping systems.
